# Non-mulberry Silk Fibroin Biomaterial for Corneal Regeneration

**DOI:** 10.1038/srep21840

**Published:** 2016-02-24

**Authors:** Sarbani Hazra, Sudip Nandi, Deboki Naskar, Rajdeep Guha, Sushovan Chowdhury, Nirparaj Pradhan, Subhas C. Kundu, Aditya Konar

**Affiliations:** 1 Department of Veterinary Surgery & Radiology, West Bengal University of Animal & Fishery Sciences, Kolkata-700037, West Bengal, India; 2 CSIR-Indian Institute of Chemical Biology, Kolkata-700032, West Bengal, India; 3 Department of Biotechnology, Indian Institute of Technology, Kharagpur, Kharagpur-721302, West Bengal, India

## Abstract

Purpose: Successful repair of a damaged corneal surface is a great challenge and may require the use of a scaffold that supports cell growth and differentiation. Amniotic membrane is currently used for this purpose, in spite of its limitations. A thin transparent silk fibroin film from non-mulberry *Antheraea mylitta* (Am) has been developed which offers to be a promising alternative. The silk scaffolds provide sufficient rigidity for easy handling, the scaffolds support the sprouting, migration, attachment and growth of epithelial cells and keratocytes from rat corneal explants; the cells form a cell sheet, preserve their phenotypes, express cytokeratin3 and vimentin respectively. The films also support growth of limbal stem cell evidenced by expression of ABCG2. The cell growth on the silk film and the amniotic membrane is comparable. The implanted film within the rabbit cornea remains transparent, stable. The clinical examination as well as histology shows absence of any inflammatory response or neovascularization. The corneal surface integrity is maintained; tear formation, intraocular pressure and electroretinography of implanted eyes show no adverse changes. The silk fibroin film from non-mulberry silk worms may be a worthy candidate for use as a corneal scaffold.

Loss of corneal transparency is a leading cause of visual impairment and blindness^1^. A major underlying pathology attributing to this condition is limbal stem cell loss or deficiency, leading to impaired re-epithelialization^2^. Developing efficient methods to obtain re-epithelialization is essential in order to prevent infections and complications. Various approaches are being tried for corneal epithelial reconstruction, such as delivery of cultured epithelial cells to the corneal surface, using different biological platforms like fibrin glue^3^,^4^, carrier-free cell sheets^5^,^6^,^7^ and amniotic membrane^8^,^9^. Although the amniotic membrane is most widely used^10^,^11^, some limitations with its usage are inadequate availability, possibility of disease transmission where rigid testing system for sterility is lacking, and amniotic membrane being semi transparent affects light transmittance during the repair phase. Another major concern is lack of standardization of methods used for denudation of Human Amniotic membrane (hAM) which is essential to support stem cell growth, although a recently published method^12^ seems to eliminate this hurdle. Thus, there is a need to identify suitable carrier for corneal epithelial cells for effective and safe transplantation to the cornea.

One option is the use of silk protein, which is being studied intensely as a potentially implantable biomaterial owing to its suitable properties: it has good tensile strength, easy handling, fast and cheap processing, high availability, controllable dimensional and material characteristics, promotes cell growth, is sterilizable, transparent, and does not require to be maintained in cold chain^13^,^14^. Much is already known of its *in-vivo* acceptance^15^,^16^,^17^,^18^.

Silk fibrion, an insoluble protein from the mulberry silkworm *Bombyx mori* (Bm) has shown some promise as a substitute for amniotic membrane. Transparent thin silk films prepared from this mulberry silk support the generation of a corneal epithelial cell sheet *in vitro*^19^,^20^,^21^, although the cellular growth is limited compared to denuded amniotic membrane^19^. New strategies are being explored for optimization of design and modification of its composition to make it more suitable in a clinical context. RGD (Arg-Gly-Asp) coupling of Bm films has led to improved cell adhesion and growth^22^,^23^. A very recent study which identified fibroin derived from the wild silkworm *Antheraea pernyi* (APSF) as an alternative choice to Bm, considering it possessed better cell adhesion motifs, failed to show improved cell growth^24^. Moreover, technical problems were encountered while preparing transparent films.

Indian non-mulberry tropical tasar silkworm *Antheraea mylitta* (Am) silk contains a natural RGD sequence and has a high mechanical strength, nutrient and metabolite permeability, elasticity, stability, and cytocompatibility^25^,^26^,^27^,^28^; it therefore possesses suitable properties to be developed as an alternative biomaterial for ophthalmic use^29^. However, an important prerequisite for future ophthalmic application is to evaluate its biocompatibility in the eye.

As we know of no previous report on the evaluation of silk fibrion from *Antheraea mylitta* for growth of corneal cells or the response of the eye, we therefore studied the suitability of non-mulberry silk films for support of corneal cell growth and preservation of cellular characteristics, and compared the cell growth with that on amniotic membrane. We further evaluated its biocompatibility and effect on ocular physiology after intra-stromal implantation in rabbit corneas.

## Results

### Transparency and Refractive index of the silk films

**Figure 1:**
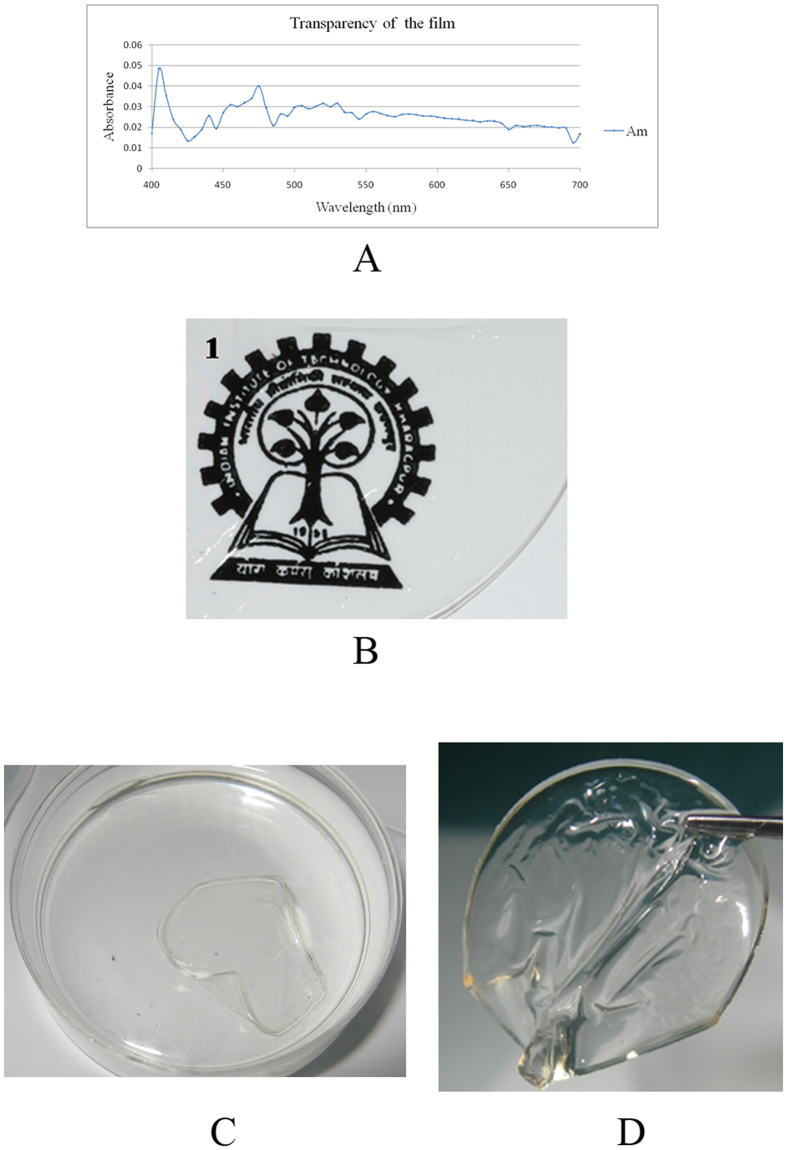
Determination of transparency of Am films. Absorbance spectra of transparent thin films of *Antheraea mylitta* (Am) fibroin were generated using a Multiskan spectrum. Scanning was performed from 400 to 700 nm wavelength range with 5 nm intervals. The graph (**A**) shows that the film has an absorbance of 0.01 – 0.04 in the visible range (400–700 nm); transparency was calculated using Beer’s law. Visual appearance of 2D transparent thin film made of Am fibroin, the image can be clearly visualized through the transparent film (**B**). Films were very thin with smooth surface (**C**) and easy to handle (**D**).

Am films produced with fibroins from the non-mulberry, Indian tropical tasar silkworm, have a light absorbance of 0.01–0.04 in the visible range (400–700 nm). The percentage transmittance was calculated to be 94.4 ± 0.006%. The mean ± s.d. of refractive index was determined to be1.44 ± 0.03. The transparency (Fig. 1) as well as the refractive index makes the films suitable candidates for use as a corneal scaffold.

### Thickness of Films as determined by histological sections

The average thickness of the films (n = 15) was evaluated as 30 ± 9.7 μm.

### Antheraea mylitta silk films for generation of a corneal cell sheet

**Figure 2:**
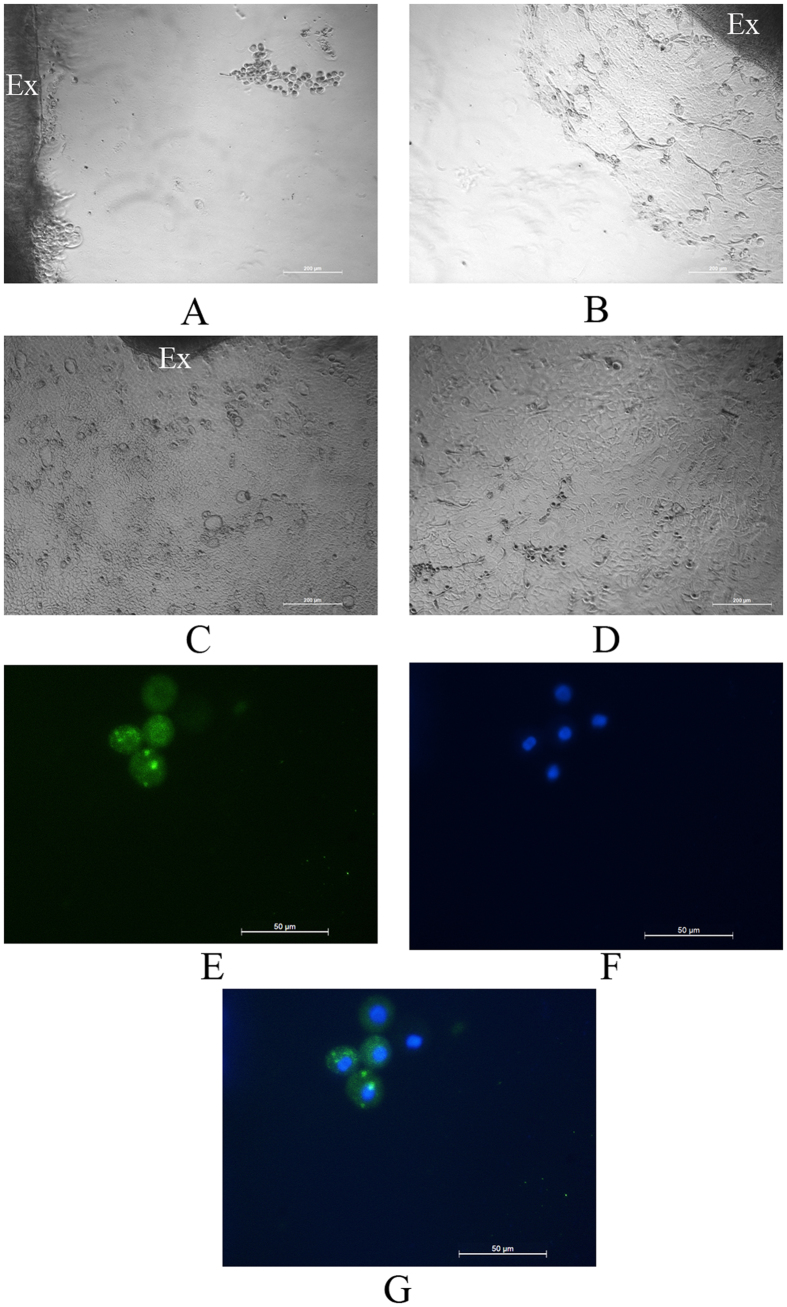
Effect of Am films on corneal epithelial cell growth and characteristics: Corneal explants (marked as Ex) were cultured on Am films, sprouting of epithelial cells were observed on day 4 (**A**), cellular outgrowth continued to occur and cells had grown further on day 6 (**B**) and gradually formed a cell sheet on day 10 with corneal epithelial cells displaying cobble stone appearance (**C**). Corneal epithelial cells cultured on culture discs also showed similar phenotype (**D**). Immunocytochemistry showed expression of cytokeratin3 by the cells (**E**), nuclei stained with DAPI (**F**) and merged image (**G**).

**Figure 3:**
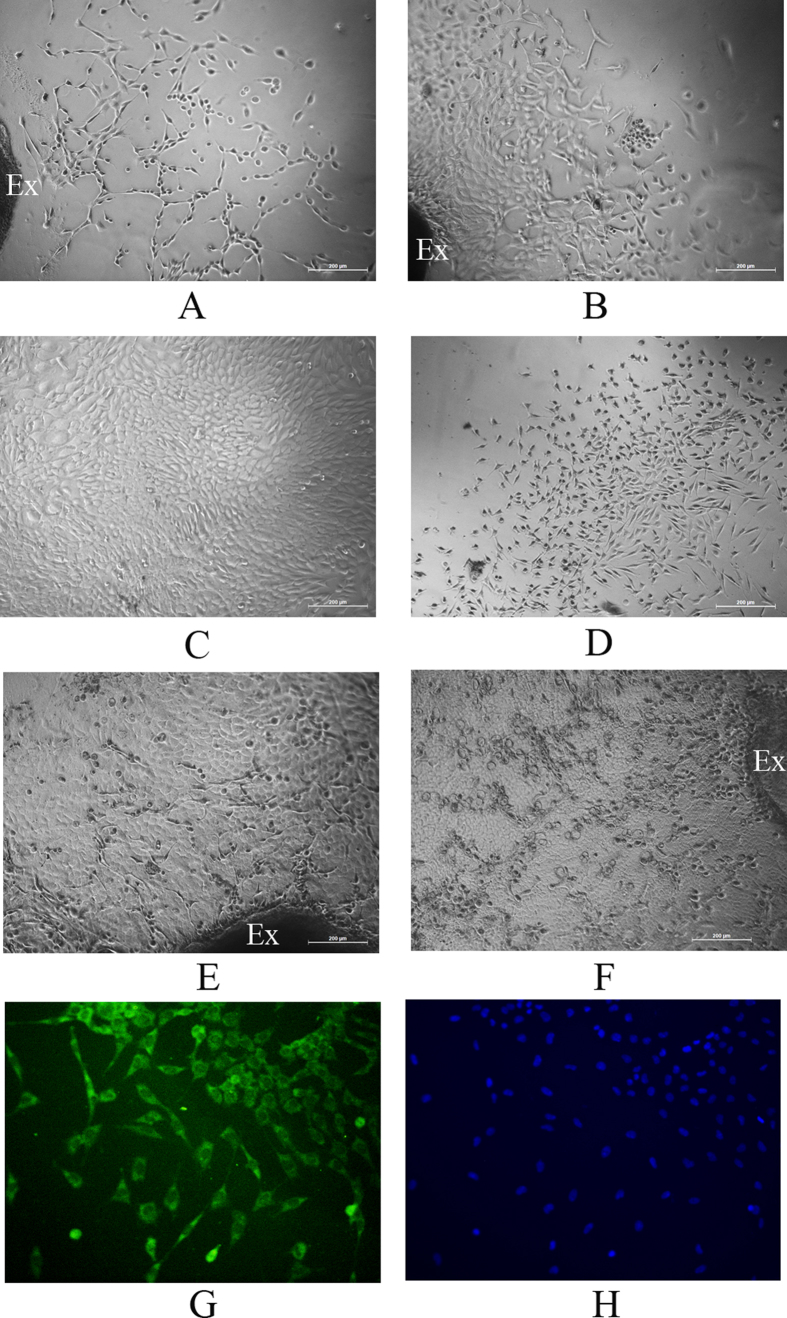
Effect of Am films on growth of keratocytes. Corneal explants (marked as Ex) were cultured on Am films, sprouting of keratocytes from the implant were observed on day 4 (**A**), the cellular growth increased on day 6 (**B**) and generated a cell sheet on day 10 with flattened dendritic shaped keratocytes (**C**). Keratocytes grown on culture discs (**D**). Am films also supported growth of epithelial cells over the existing keratocyte cell layer (**E,F**). Keratocytes expressed vimentin (**G**), nuclei stained with DAPI (**H**).

**Figure 4:**
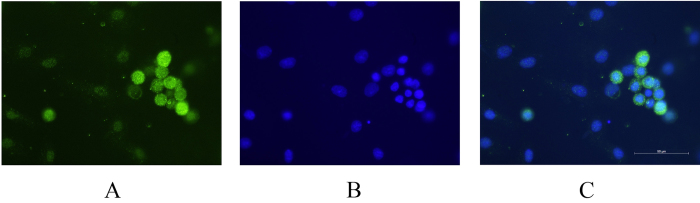
Growth of corneal cell on Am silk film and amniotic membrane. Corneal explants were cultured on Am silk film (**A**) and amniotic membrane (**B**) and cellular outgrowth were imaged on day 8, the percentage of image area covered by outgrowing cell on Am silk films and amniotic membrane were determined by image J software and compared (**C**). *Data is presented as mean* *±* *sd, N* *=* *15.*

Corneal explants obtained from rat cornea were cultured on the film to study whether it can support cell division, migration, attachment and proliferation. On day four, sprouting of epithelial cells and keratocytes from the implant was observed, which migrated and attached to the film (Figs 2A and 3A). These cells further divided and gradually covered a substantial portion of the film (Figs 2B and 3B) and ultimately formed a cell sheet (Figs 2C and 3C). The epithelial cells and keratocytes were well differentiated with their cobble-stone (Fig. 2C) and flattened dendritic (Fig. 3C) appearances, respectively, and did not differ phenotypically from the cells cultured on the tissue culture plates (Figs 2D and 3D). Interestingly, cells were also observed to be growing in bilayers over films (Fig. 3E,F). Expression of cytokeratin3 in corneal epithelial cells (Fig. 2E,F DAPI, 2G merged) and vimentin in keratocytes (Fig. 3G,H DAPI) were detected by immunocytochemistry; therefore, the corneal cells on the film preserved their normal physical as well as biological characteristics. Immunocytochemistry also showed expression of ABCG2 in cells originating from the limbal explants on the films (Fig. 4) which indicates Am films support growth of limbal stem cells.

### Comparison of cell growth on Am silk films and amniotic membranes

**Figure 5:**
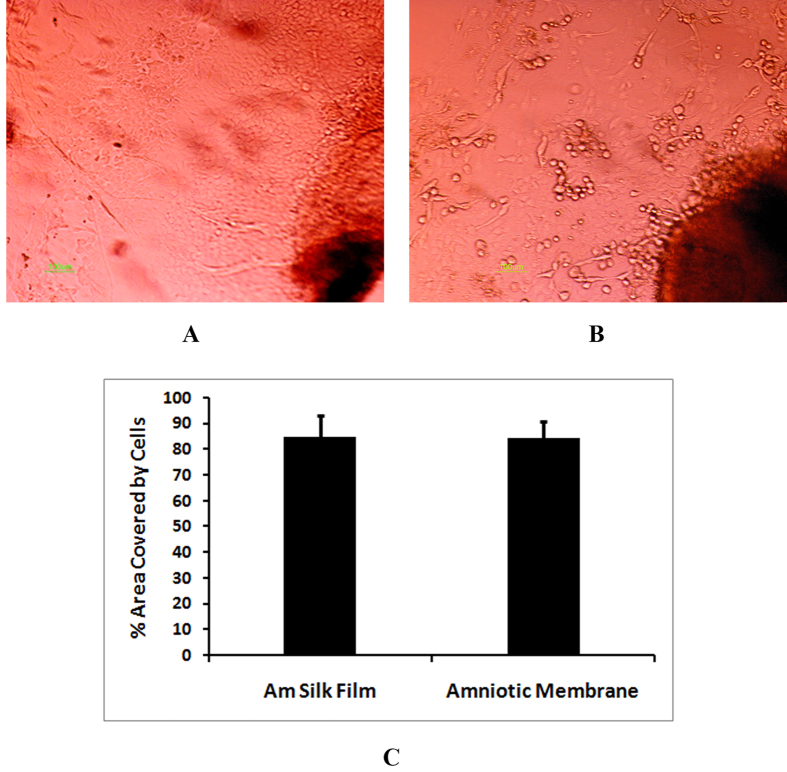
Support of Am films for limbal stem cell growth. Limbal explants were grown on Am silk films, immunocytochemistry showed expression of ABCG2 from the growing cells (**A**), nuclei stained with DAPI (**B**), and merged image (**C**).

Outgrowth of cells from the corneal explants onto Am silk films was compared with the outgrowth on amniotic membrane. The percentage area covered by outwardly migrating cells on Am silk films (84.96 ± 8.5, n = 15) and amniotic membrane (84.58 ± 6.4, n = 15) did not differ significantly (p > 0.05) (Fig. 5).

### Clinical response of the eye following implantation

**Figure 6:**
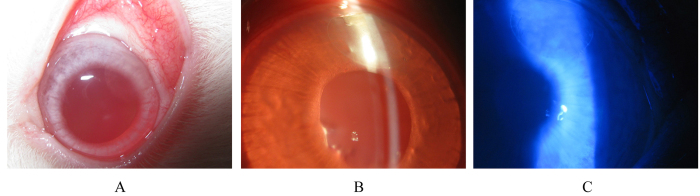
Implantation of Am films within the cornea. Gross examination (**A**) and slit lamp biomicroscopy (**B**) shows that the film implanted within the cornea remained stable and transparent after 2 months and the films were well tolerated *in-vivo*. Absence of fluorescein dye shows corneas were free from ulceration and erosion (**C**).

Am silk films were implanted in corneal pockets of 6 rabbits, and the clinical behavior was compared with corneas that underwent sham-surgery. There was no sign of uveitis or keratitis in any of the eyes with either implants or sham surgery. The gross (Fig. 6A) as well as slit lamp biomicroscopic (Fig. 6B) images shows clear transparent implants within the cornea. The corneas remained clear and were free from neovascularization. The iris was clearly visible through the film under the slit lamp biomicroscope. Clinically, the films looked intact after two months of implantation.

### Corneal surface integrity

The corneal surface integrity was maintained. All eyes were negative in the fluorescein dye test at 7 days, 14 days and 2 months after surgery (Fig. 6C)

### Tear production

Tear production was measured following film implantation and compared with the preoperative and sham-operated eyes. The tear production at day 7 (10.6 ± 1.2 mm/min), day 14 (11.3 ± 1.2 mm/min) and 2 months (9.7 ± 1.6 mm/min) after implantation did not vary significantly (p > 0.05) from the preoperative values (10.7 ± 1.0 mm/min) or the sham-operated eyes at 2 months (9.7 ± 2.1 mm/min).

### Intraocular pressure

Intraocular pressure in the eyes after 7 days (11.0 ± 0.9 mmHg), 14 day (9.0 ± 0.9 mmHg) and 2 months (10.3 ± 1.9 mmHg) following film implantation did not vary significantly (p > 0.05) from either the sham-operated eyes after 2 months (11.0 ± 2.2 mmHg) or from the normal preoperative eyes (10.2 ± 0.7 mmHg).

### Electrophysiology of the eye

Two months after film implantation, ERGs from six eyes with implants were compared to the contra-lateral control eyes to evaluate any changes in retinal physiology. The mean ± standard error of the mean of the scotopic as well as photopic b-wave amplitudes and implicit times elicited by different stimulus intensities did not differ significantly (p > 0.05) from the control eyes (Table 1).

### Corneal histology

**Figure 7:**
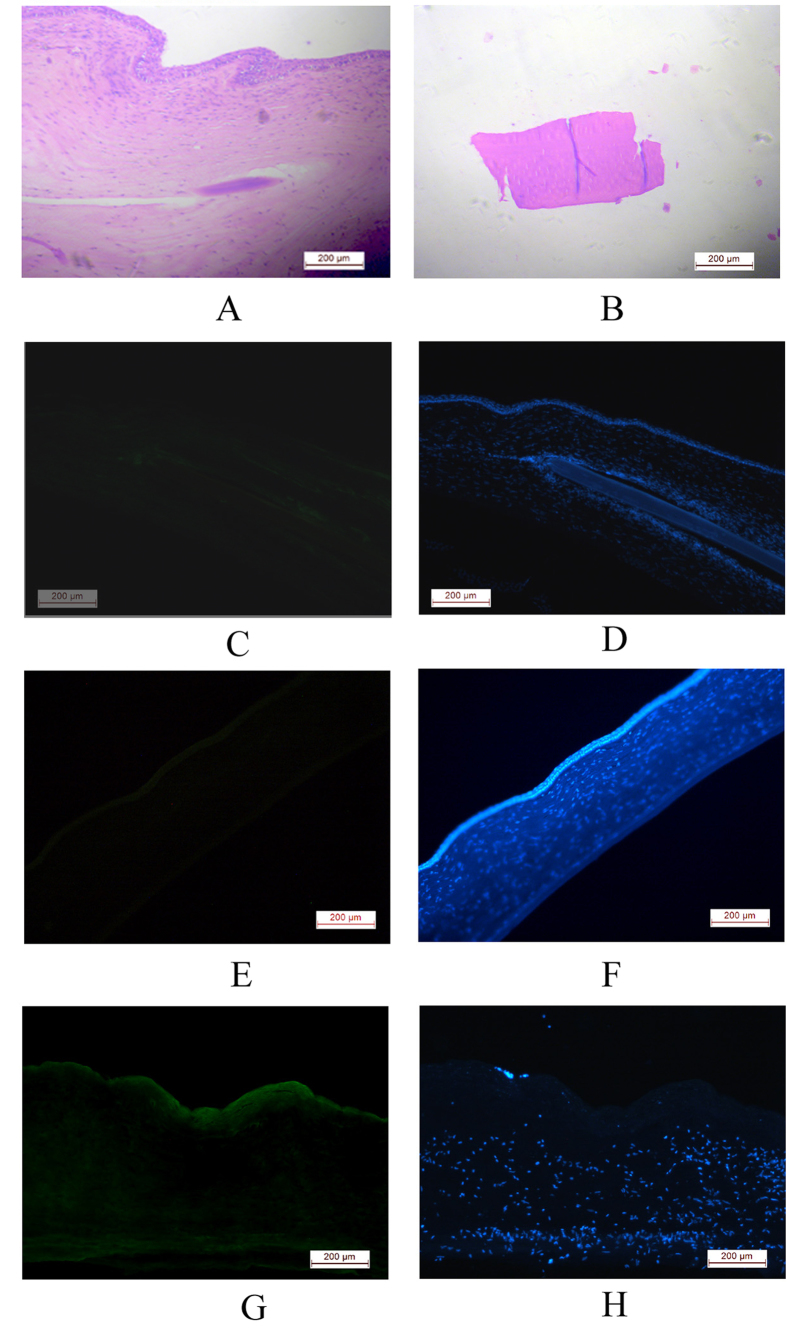
Effect of Am films on corneal histology following intra stromal implantation. Histopathology shows Am films within the corneal section, and the cornea maintained their normal cellular architecture (**A**). The films did not degrade after 2 months (**B**). Immunohistochemistry of the sections from film implanted corneas ((**C),D**, DAPI) like the normal corneas ((**E,F**, DAPI), did not express α-SMA, whereas alkali burnt corneas expressed α-SMA ((**G,H**, DAPI).

Histological sections of eyes carrying implanted films show the implant within the cornea (Fig. 7A). The normal architecture of the cornea was preserved. The corneas were devoid of any necrotic and degenerative changes that confirmed the biocompatibility of the implant. Absence of any inflammatory reaction and neovascularization indicated its acceptability within the cornea (Fig. 7A). The film remained intact within the cornea (Fig. 7B). We performed immunohistochemistry on tissue sections from the Am film implanted cornea using α-SMA to determine if there is any myofibroblast formation which is mainly responsible for fibrotic changes and did not observe expression of α-SMA (Fig. 7C). The staining resembled the normal cornea (Fig. 7E). This indicated absence of any fibrotic changes in the implanted cornea. Alkali burn induces EMT in corneal cells and cause fibrosis of the cornea, therefore we used sections from alkali burnt rabbit cornea as a positive control for the antibody, and could detect expression of α-SMA (Fig. 7G).

### Ultrastructure of cornea

**Figure 8:**
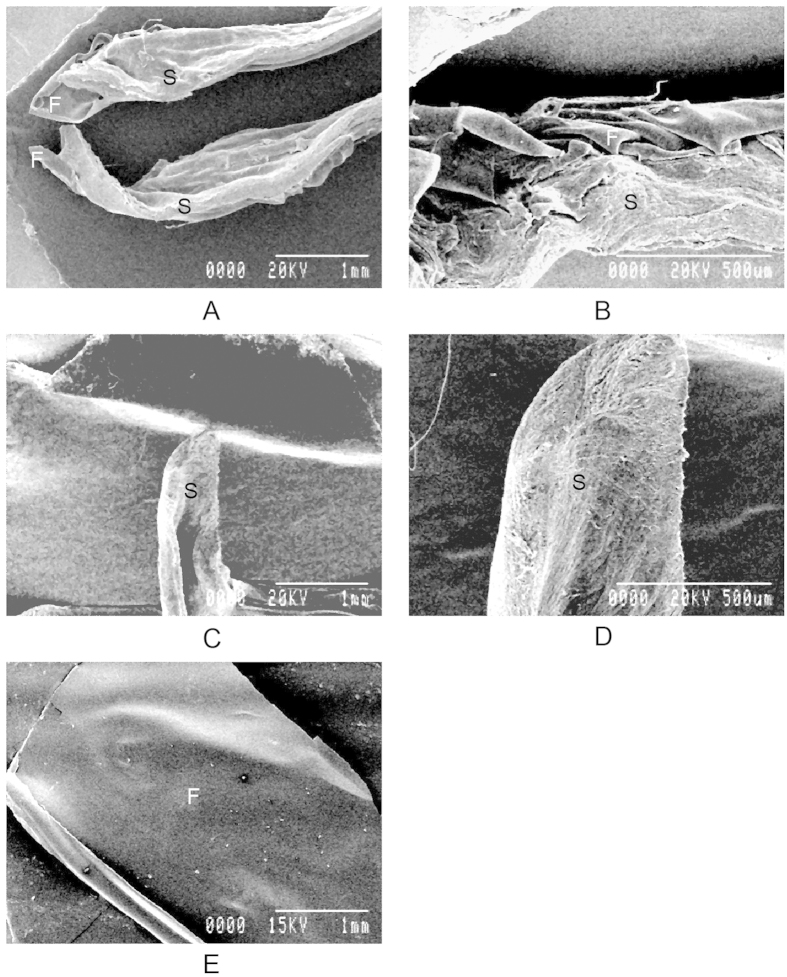
Ultrastructure of the cornea. Scanning electron microscopy shows that Am films (marked as **F**) within the cornea remained intact, corneal stroma (marked as S) attached to the membrane retained its normal architecture (**A,B**), similar to sham operated corneal stroma (**C,D**). Am films (**E**).

Scanning electron microscopic images of the film, normal corneas and film-implanted corneas are presented in Fig. 8. The image of an implanted cornea clearly shows normal corneal stroma around the film. The corneal architecture was similar to the cornea of sham-operated eyes. The silk implants within the cornea remained intact.

## Discussion

Keratitis, trauma, chemical injury and other diseases result in irreparable loss of corneal tissue and may heal leaving a scar or distorted host tissue that culminates into partial or complete vision loss. Corneal transplantation is the only treatment option but can often not be performed due to lack of human donor corneas. Several different materials have been used to develop artificial corneal substitutes, such as biosynthetic implants from human recombined collagen type 1^30^ or acellular porcine cornea^31^,^32^ but they often pose expensive and complex alternatives for clinical use. Efforts to develop more affordable corneal prosthetic material have identified fish scale derived collagen matrix as a promising biomaterial^33^. Despite its drawbacks, amniotic membrane continues to be used for corneal repair. Efforts to develop more suitable biomaterial as substitute for cornea identified silk fibroin as a potential graft material^21^. Some progress has been made towards the development of a silk-based corneal scaffold using Bm fibroin^19^,^20^,^21^,^22^,^23^. Bm films have been reported to only support limited cell growth^19^. Improvement of porosity by casting Bm fibroin film in the presence of a low molecular weight poly ethylene glycol (PEG molwt = 300) supported the generation of a cell sheet^34^. The corneal epithelial cells were grown in the presence of 3T3 feeder cells that provide growth factor support. However, this study did not report epithelial cell growth in absence of feeder cells.

In our study, we report for the first time that corneal stem cells, epithelial cells as well as keratocytes can grow on the Am film, leading to a confluent cell sheet. We compared the cell growth with that on amniotic membrane and not with traditional variety of silk i.e. *Bombyx mori* since it is already the standard scaffold in clinical use, whereas *Bombyx mori*, though promising, is still in investigational stage. Interestingly, our results also show that the cell growth on Am films was comparable to the outgrowth on amniotic membrane. The presence of an RGD sequence in Am may have contributed to robust cellular attachment and thus supported confluent cell growth^29^. Previous studies also report that RGD coupling of Bm silk films enhanced corneal cell growth *in vitro*, compared to films without RGD coupling^22^,^23^. The Am film also supports simultaneous growth of epithelial cells and keratocytes on the same membrane, which essentially resembles the *in vivo* requirement. In an attempt to mimic a partially-damaged cornea, instead of separating the cells and culturing on the membrane, we placed the corneal explants on the membranes. We observed cell budding from the explants, and migration of cells and attachment to the membrane, following which, the cells gradually covered the membrane with an epithelial cell sheet as required to fill the denuded epithelium. Most importantly, we found Am silk films to support growth of both ABCG2 positive corneal stem cells, differentiated corneal epithelial cells with a cobble-stone appearance which expressed cytokeratin3 and flattened dendritic keratocytes expressing vimentin. This raises the possibility of using Am silk fibroin as a scaffold for delivery of corneal stem cells and differentiated epithelial cells and keratocytes to the cornea. In our *in-vivo* investigation, the intrastromal implants of Am films incited no adverse inflammatory reaction or neovascularization. They were well tolerated by the rabbit eyes. It was already known that placement of silk film in a buccal mucosal defect does not show any local or systemic immunological incompatibilities^35^. There was no fibrotic change within the implanted cornea. The films maintained transparency enabling clear visualization of iris during the study period. Histopathology and scanning electron microscopy also confirmed the absence of inflammatory cells. Similarly, an *in vivo* study showed that the Bm film is biocompatible with the rabbit cornea^34^. Our studies with Am films using additional parameters on ocular physiology showed that there is no dysregulation of tear formation, aqueous flare, or change in intraocular pressure following intrastromal implantation, all of which are essential components for assessing optimum corneal homeostasis. As an extra control, we determined that the Am films do not change the retinal function as shown by ERG studies.

Refractive index of the silk films obtained in our study i.e. 1.44 corroborates with previous studies^36^ and most importantly matches with refractive index of corneal epithelium or stroma which were determined as 1.4, 1.38 respectively^37^.

In our study Am films remained intact within the cornea for the study period of two months, previous study has reported stable silk films within the cornea for longer periods i.e. 6 months^38^. In the biological system silk protein is affected by proteases, however different types of silk provide variable rates of biodegradation^39^,^40^. The Am films contain a considerable amount of β-sheets that probably provide its stability^41^. Interestingly, the degradation of the silk films can be tailored by modifying the β-sheet content^42^. This makes them suitable for corneal reconstruction, as it provides an option to tune the degradation of the films to match the normal regeneration of the cornea. Silk films can therefore have potential utility in corneal reconstruction and regeneration.

## Conclusion

Silk fibroin films from non-mulberry *Antheraea mylitta* provide suitable physical characteristics, support corneal cell growth and are biocompatible in rabbit eyes. Films display the potential to be used as a matrix for corneal reconstruction and the delivery of new cells to the cornea. Its response following implantation within the limbus and other intraocular sites and influence on corneal nerve regeneration warrants further study before clinical use.

## Materials and Methods

### Preparation of regenerated silk fibroin solution

Silk protein fibroin was extracted after isolation of the silk glands from the fully grown 5th instar live larvae of Indian tropical tasar, *Antheraea mylitta* (Am) collected from our IIT Kharagpur Biotechnology Farm. Hydrophobic fibroin protein was isolated in distilled water by squeezing the posterior glands with fine tweezers. The isolated crude protein was then processed according to the protocol described earlier^43^. In summary, the protein was dissolved using aqueous 1% sodium dodecyl sulfate (SDS) buffer containing TRIS (10 mM and pH-8.0) and EDTA (5 mM). For removal of excess SDS, the solution was dialyzed extensively using a 12 kDa MWCO (Sigma, St. Louis, USA) membrane against de-ionized water for 10 hrs and the removal of SDS was confirmed by determining the bound and unbound SDS concentration using colorimetric method (OD 651 nm)^44^ and fluorometric assay^43^.The concentration of the dialyzed protein was measured using the Bradford method. The silk solution was kept at 4 °C for a week until films are made.

### Fabrication of film (solvent evaporation)

The regenerated 2% (w/v) fibroin solution was used for fabricating the films; 150 μl was cast on the surface of each well-cover on the inner side of a 24-well tissue culture plate (TCP) cover. The plates were then left inside the laminar hood with airflow overnight to dry. Dried transparent films were collected in 70% alcohol and stored for future use.

### Transparency of the films

Transparency of the silk protein fibroin films was measured in terms of absorbance in the visible range using a Multiskan Spectrum (Thermo Electron Corporation). Films were scanned from 400 to 700 nm wavelength range with 5 nm intervals. The transparency of the films was calculated in terms of percentage of transmittance using Beer’s law.


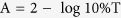


(where A: absorbance and T: transmittance)

### Refractive index of thin Am films

Refractive index of Am films were determined by ellipsometry using a Woollam M2000D ellipsometer (J. A. Woollam Co., Inc., Lincoln, NE) between 190 nm and 998 nm aligned at a nominal incidence angle of ≈ 70° from the surface normal. The ellipsometric thickness and the exact incidence angle were separately determined prior to obtaining the data. The reported data are the averages of three separate measurements on each sample.

### Determination of thickness of Am films

Thickness of the Am films were determined prior to implantation, cryo sections of 15 different Am silk films were made, fixed on 0.5% gelatin-coated slides, stained with hematoxylin-eosin and thickness of the films was measured under a microscope using scale bar.

### Corneal explant culture on silk film and amniotic membrane

Corneal cells were grown from the corneal explants using standard method^45^ with necessary modification. For culture of limbal stem cell and corneal epithelial cells, corneas were excised along with corneo-scleral junctions from young Sprague Dawley rats and cut into triangular-shaped wedges, using a single cut of the scalpel, placed on the silk films and denuded amniotic membranes (Ramayamma International Eye Bank, LVPEI, Hyderabad, India) kept on separate 35 mm culture plates (Nunc) with epithelial side up. Fifty micro liter of Epilife [cat no. MEPI500CA, Gibco] containing Human corneal growth supplement (1 μl/ml) [cat no. S0095, Gibco], 7% FBS [Gibco] and antibiotic mixture (50 IU/ml penicillin and 50 μg/ml streptomycin) (Cat. No.15140, Gibco) was carefully placed upon each segment and the plates were incubated overnight (37 °C, 5% CO_2_). The next day, 1 ml of complete medium was added to each plate. Media was changed every three days. Explants for keratocytes were cultured in a similar manner after removing the epithelial layer with a sterile scalpel, and using Stemline^TM^ Keratinocyte Medium (Sigma, S0196) containing human epidermal growth factor (0.1 ng/ml, Sigma), insulin (5 μg/ml, Sigma), hydrocortisone (0.5 μg/ml, Lonza), bovine pituitary extract (30 μg/ml, Sigma) and antibiotic mixture (50 IU/ml penicillin and 50 μg/ml streptomycin) (Cat. No.15140, Gibco). Films were examined under a microscope (ECLIPSE TE 2000, Nikon, Japan) and imaged on the days as indicated. Immunocytochemistry was performed with ABCG2, cytokeratin3 and vimentin antibody. For comparison of cell growth between Am silk film and amniotic membrane, cellular outgrowth on the Am films and amniotic membrane from the explants were imaged on day 8 and the image area covered by cells were calculated as a percentage of total image area by ImageJ software from 15 explants on each type of membrane and presented as mean ± sd.

### Immunocytochemistry

Medium was aspirated and the films were rinsed in phosphate-buffered saline (PBS) and fixed with 4% paraformaldehyde for 20 minutes at 4 °C. Then the cells were permeabilized with 0.1% Triton X-100 for 10 min on ice. After rehydration in PBS and air drying, the films were blocked with 3% goat serum (Abcam) in PBS for 2 hours. Then primary antibody for cytokeratin 3 (1:100 in 3% Goat Serum; Millipore, #CBL218-I) was added to the membrane and kept in a humidified chamber overnight at 4 °C. Films were washed thrice for 15 minutes in PBS-T (Phosphate-Buffered Saline/Tween-20); Alexa Flour 488 (Invitrogen)-conjugated secondary antibody (1:500 in 3% goat serum) was added to each membrane and incubated for 60 minutes at 37 °C. After washing, films were counterstained with DAPI nuclear stain and mounted using mounting medium for fluorescent microscopy (DAKO), and sealed under a cover slip. The cells were viewed under a fluorescence microscope (ECLIPSE TE 2000, Nikon, Japan). Immunocytochemistry was also performed using ABCG2 (1:100 Sigma, AV 43649), vimentin (1:100, Santa Cruz Biotechnology, Sc-6260) primary antibody and Alexa Flour 488 (Invitrogen)-conjugated secondary antibody (1:500 in 3% goat serum) and viewed under a fluorescence microscope (DMI 4000B; Leica Microsystems).

### Animal experimentation

This study was specifically approved (Approval No. EC- 67,dated 21-5-13) by the “Institutional Animal Ethics Committee” registered with CPCSEA (Registration no 763/03/a/CPCSEA, dated 05.06.2003), Department of Pharmacology & Toxicology, Faculty of Veterinary& Animal Sciences, West Bengal University of Animal and Fishery Sciences, Kolkata, India. The work followed the guidelines of the Association for Research in Vision and Ophthalmology (ARVO).

The study was performed in 6 New Zealand White rabbits of either sex, around 1 year of age. The eyes were examined and screened for preexisting anomalies. Surgery was performed under general anesthesia^46^ with a combination of xylazine hydrochloride (Xylaxine®; Indian Immunologicals Ltd., Hyderabad, India) @ 5 mg/kg and ketamine hydrochloride (Ketamine 50®; Themis, Haridwar, Uttarakhand, India) @ 35 mg/kg, and topical instillation of 0.5% proparacain hydrochloride (Paracain^©^ Sunways India Pvt Ltd. Mumbai), tropicamide eye drops (Tropicacyl®, 1% w/v; Sunways (India) Pvt. Ltd., Mumbai, Maharastra, India) and phenylephrine eye drops (Ocurest® 0.12%; Centaur Pharmaceuticals Pvt. Ltd., Mumbai, India). A partial lamellar dissection was performed, using a 1 mm bevel up-crescent knife to create the initial corneal incision 1 mm from the limbus. Hereafter, a lamellar dissector was used to create the stromal pocket in the cornea of both eyes. Am silk films were washed 3 times 15 minutes each in PBS containing 50 IU/ml penicillin and 50 μg/ml streptomycin^47^. Using a 4 mm diameter trephine, the films were cut on a sterile teflon block and in order to prevent wrinkling 25 μl of viscoelastic (Appavisc® 2% w/v; Appasamy ocular devices, Pvt. Ltd. (Pharma division), Vadamangalam, Puducherry, India) was put on the film, after which the film was inserted into the stromal pocket in one eye of each rabbit. No sutures were placed and the incision was left for self healing. Post operatively, all eyes received ciprofloxacin eye drops 3 times daily for 7 days to prevent infection. The following tests were conducted for assessment of the host response to the implant.

### Slit lamp biomicroscopy

To evaluate the clinical response of the cornea as well as the anterior segment and also to monitor the implants within the cornea, the eyes were examined under a slit lamp biomicroscope daily for one week, followed by once a week for the rest of the study period of 2 months.

### Fluorescein dye test

To ensure corneal surface integrity and detect any corneal defect following implantation of the films, a fluorescein dye test was conducted on days 7, 14 and at 2 months post surgery. For this, a paper strip impregnated with dry orange fluorescein dye was wetted and touched to the tear pool inside the lower conjunctival sac of the eye. The dye was then washed away with normal saline. The eyes were examined with a slit lamp biomicroscope under a blue filter to detect the natural green fluoresce observed with retention of dye in eyes with corneal defects.

### Schirmer tear test

To evaluate if the implantation of an Am silk film affects tear production, Schirmer’s test was performed in all eyes on the 7th and 14th day post surgery and after 2 months. The Schirmer’s test strip was placed in the culdesac of the medial canthus in each eye and the wetting for one minute was noted and compared.

### Intraocular pressure

Intraocular pressure was measured preoperatively and on days 7, 14 and at 2months after surgery to ascertain that implants did not affect the intraocular pressure. The eyes were desensitized with a topical anesthetic, 0.5% proparacaine hydrochloride and the intraocular pressure was measured with a Tono-pen® (Reichert, USA). An average of three readings per eye was taken at each time point.

### Electroretinography (ERG)

Acute inflammatory response of cornea may lead to retinal gliosis^48^ which may alter retinal physiology; we therefore performed ERGs to evaluate any such effect following Am silk film implantation. Electroretinography was performed two months after implantation following standard procedure as described earlier^49^. In brief, the rabbits were dark adapted for 2 hours, mydriasis was achieved using 1% topicamide and 2.5% phenylephrine; rabbits were anesthetized with a combination of xylazine hydrochloride @ 5 mg/ Kg and ketamine hydrochloride @ 35 mg /Kg body weight and 0.5% proparacain hydrochloride was used for topical anesthesia. 50 μl of viscoelastic was applied on the cornea and ERG recording was conducted following the ISCEV guidelines (Reti Animal, Roland Consultancy, Friedrich-Franz, Brandenburg-an der Havel, Germany).

### Tissue collection

At the end of 2 months, the animals were sacrificed and eyes were enucleated for histology. The corneas of all eyes were removed and divided into two equal halves and preserved in 2.5% glutaraldehye or 10% formaline for scanning electron microscopy and histopathology, respectively.

### Animal model for corneal fibrosis

One eye of a single New Zealand white rabbit was treated with 0.5 N NaOH for 30 seconds under general anesthesia with Xylazine hydrochloride and Ketamine hydrochloride as described above. The cornea was anesthetized with 0.5% proparacain hydrochloride and mydriasis was achieved with topical drops of tropicamide. After 30 seconds, the eye was rinsed with normal saline, antibiotic drops were instituted; the animals were sacrificed after 7 days and the corneas harvested.

### Histopathology

Corneas were fixed in 10% formaldehyde, dehydrated in graded alcohol, embedded in paraffin and cut into 5 μ sections. Sections were stained with hematoxylin-eosin and examined under a microscope (Leica DM 2500).

For immunohistochemistry, corneas were fixed in 4% paraformaldehyde overnight at 4 °C, washed in PBS, transferred to 30% sucrose in PBS and kept at 4 °C until the corneas sink. Corneas were fixed in OCT gel (Optimal cutting temperature, an inert support medium) and froezen at −26 °C. Sections of 15 μ were obtained at −26 °C and fixed on 0.5% gelatin-coated slides.

### Immunohistochemistry

Sections were rehydrated and permeabilized with 1% triton-X for 20 minutes. Nonspecific binding was blocked by placing the sections in 6% BSA for 4 h in a humidified chamber. Samples were washed three times in PBST containing 2% BSA for 5 min, incubated overnight in a humidified chamber with α-SMA primary antibody (1:50, Abcam, ab66133), and washed three times with PBST containing 2% BSA for 5 min. The slides were incubated in a humidified chamber with an anti-rabbit secondary antibody tagged with Alexa Fluor (1:100, A10042; Invitrogen, Carlsbad, CA) for 2 h, and washed three times with PBST containing 2% BSA for 5 min. The sections were stained with DAPI (4′–6-diamidino-2-phenylindole). The immune complexes were examined under a fluorescence microscope (Leica DM 2500; Leica) and imaged.

### Scanning Electron Microscopy

Scanning electron microscopy of silk films, sham-surgery and normal corneas and silk- implanted corneas was performed as described earlier^46^. In brief, tissues preserved in 2.5% glutaraldehyde were dehydrated in graded alcohol, critical point dried, coated with gold (IB-2 ion coater; Eiko Engineering, Ibaraki, Japan), and observed under a scanning electron microscope (SEM; Hitachi S530; Hitachi, Tokyo, Japan).

### Statistical Analysis

All values, unless otherwise stated, are expressed as mean ± s.d. The minimum number of replicates for all measurements was 6. Comparison between groups was done using One-way analysis of variance and the significance level was set at p < 0.05.

## Additional Information

**How to cite this article**: Hazra, S. *et al.* Non-mulberry Silk Fibroin Biomaterial for Corneal Regeneration. *Sci. Rep.* **6**, 21840; doi: 10.1038/srep21840 (2016).

## Figures and Tables

**Table 1:** Electroretinograms recorded from control and film-implanted eyes 2 months postoperatively.

	b-wave implicit time (ms)		b-wave amplitude (μV)	
	Control	Implant	Control	Implant
Scotopic 0.01	61.6 ± 3.7	60.0 ± 1.6	114.58 ± 16.9	99.0 ± 17.0
Scotopic 3.0	39.0 ± 2.4	43.5 ± 3.7	156.8 ± 16.0	141.53 ± 17.9
Scotopic 10.0	40.0 ± 3.2	39.3 ± 3.4	165.4 ± 30.7	147.4 ± 29.2
Photopic 3.0	29.7 ± 0.7	29.0 ± 0.9	90.3 ± 8.0	94.0 ± 6.0
Photopic 30 Hz flicker	N1/P1		N1/P1	
	Control		Implant	
	58.9 ± 6.9		54.8 ± 7.0	

The electroretinogram values recorded two months after implantation did not vary significantly (p > 0.05) from the contra lateral control eyes. *Data expressed as mean ± standard error of the mean* (*n* *=* *6).*
